# Ibrutinib for B cell malignancies

**DOI:** 10.1186/2162-3619-3-4

**Published:** 2014-01-28

**Authors:** Aileen Novero, Pavan M Ravella, Yamei Chen, George Dous, Delong Liu

**Affiliations:** 1 Department of Medicine, Westchester Medical Center, 100 Woods Rd, Valhalla, NY 10595, USA; 2 Department of Hematology, Xiamen Zhongshan Hospital, Xiamen University, Xiamen, China; 3 Institute of Hematology, Henan Tumor Hospital, Zhengzhou University, Zhengzhou, China

## Abstract

Research over the role of Bruton’s agammaglobulinemia tyrosine kinase (BTK) in B-lymphocyte development, differentiation, signaling and survival has led to better understanding of the pathogenesis of B-cell malignancies. Down-regulation of BTK activity is an attractive novel strategy for treating patients with B-cell malignancies. Ibrutinib (PCI-32765), a potent inhibitor of BTK induces impressive responses in B-cell malignancies through irreversible bond with cysteine-481 in the active site of BTK (TH/SH1 domain) and inhibits BTK phosphorylation on Tyr^223^. This review discussed in details the role of BTK in B-cell signaling, molecular interactions between B cell lymphoma/leukemia cells and their microenvironment. Clinical trials of the novel BTK inhibitor, ibrutinib (PCI-32765), in B cell malignancies were summarized.

## Introduction

Bruton’s tyrosine kinase (BTK) is a cytoplasmic tyrosine kinase, which is essential in B-lymphocyte development, differentiation, signaling and survival [1]. The *BTK* gene is located on X-chromosome at Xq21.33-Xq22. The gene has 19 exons spanning 37.5 kb genomic DNA. It encodes a non-receptor tyrosine kinase of the Btk/Tec family. The Tec family kinases (TFKs) have five members (Btk, Tec, Itk, Txk, Bmx), forming the second largest family of cytoplasmic tyrosine kinases in mammalian cells [2]. BTK is expressed in almost all hematopoietic cells, except T-cells and plasma cells. However, its essential functions appear to be limited to B-cells. BTK is required for B-cell receptor (BCR) signaling and implicated in the development of the B-cell malignancies including chronic lymphocytic leukemia (CLL), mantle cell lymphoma (MCL), follicular lymphoma (FL), diffuse large B-cell lymphoma (DLBCL) and acute lymphocytic leukemia (ALL) [3,4].

Several preclinical and clinical studies have been performed, targeting BTK in B-cell malignancies. Ibrutinib/PCI-32765, a novel potent inhibitor of BTK induces impressive responses in B-cell malignancies and has been approved for therapy of refractory mantle cell lymphoma.

### BCR/BTK signaling pathway

**Figure 1:**
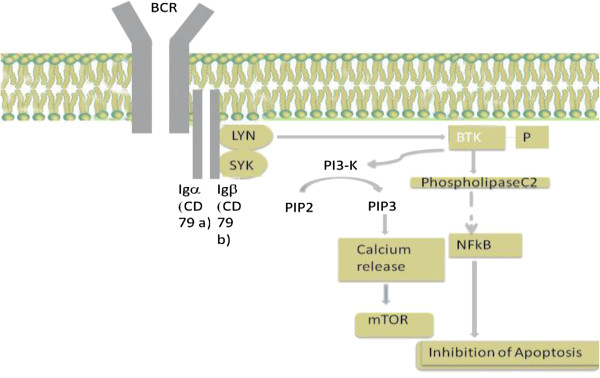
**A schematic representation of BCR/BTK signaling pathway.** The BCR consists of a transmembrane immunoglobulin (Ig) receptor associated with the Ig-alpha (CD79a) and Ig-beta (CD79b) heterodimers. The activation of BTK and PI3 kinase after antigen binding to BCR prompts calcium release, which leads to activation of the signaling cascade. BCR: B cell receptor; BTK: Bruton’s tyrosine kinase.

BCR is critical for normal B-cell development and is also associated in the development of the most common B-cell malignancies [5-7]. BCR serves as an antigen receptor and regulates multiple cellular processes, including proliferation, differentiation, apoptosis, and cell migration [2,8]. The BCR consists of a transmembrane immunoglobulin (Ig) receptor associated with the Ig-alpha (CD79a) and Ig-beta (CD79b) heterodimers [7,9-11]. Once the antigen binds to the receptor, the tyrosine kinases LYN and SYK initiate a signaling cascade that involves downstream kinases, adapter molecules, and generation of second messengers [9] (Figure 1). BTK is one of the signaling molecules that is essential in the BCR pathway. BTK comprises of several domains from the N-terminus: pleckstrin homology (PH), Tec homology (TH), SH2, SH3, and kinase (SH1) domains [1]. BTK requires Zn^2+^ for optimal activity and stability. Binding and coordination of BTK to the Zn^2+^ ion is mediated by a highly conserved zinc finger motif located in the TH domain. Mutations affecting Zn^2+^ binding lead to the generation of extremely unstable protein [12,13].

Additional signaling molecules in the BCR signaling pathway include mammalian target of rapamycin (mTOR), SYK, LYN, phosphatidylinositol 3- kinase (PI3K) [14,15], the adaptor protein GrB2. The activation of BTK and PI3 kinase prompts calcium release and activation of mTOR, protein kinase C-beta, AKT kinase, and mitogen-activated protein kinase ERK. These events then result in cell proliferation and survival of B-cells, which is mediated by up-regulation of transcription factors, mainly nuclear factor-kB (NFkB). Upon activation of BTK, PI3K is then activated, which stimulates the production of phosphatidylinositol-3, 4, 5 (PIP3) [14,15]. Once an adequate amount of PIP3 is produced, BTK is mobilized to the plasma membrane. Phosphorylation of the BTK at the Y551 site is done by the Src family kinases, especially LYN and FYN. The phosphorylated BTK activates phospholipase C2, leading to downstream activation of protein kinases, and finally activation of transcription factor NFkB. The stimulation of NFkB pathway leads to inhibition of apoptosis (Figure 1). This series of events has been associated with the proliferation and survival of B-cell malignancies [16,17]. BTK was found to be significantly overexpressed in CLL compared with normal B-cells. Although BTK is not always constitutively active in CLL cells, BCR or CD40 signaling is accompanied by effective activation of this pathway.

#### Preclinical studies

Ibrutinib is an orally active irreversible BTK inhibitor through bonding with cysteine-481 in the active site of BTK (TH/SH1 domain). It inhibits BTK phosphorylation on Tyr^223^ and has an IC_50_ of 0.5 nM for more than 24 hours [18]. In an in vitro study, Herman et al. demonstrated dose- and time-dependent cytotoxicity in CLL through caspase-3 dependent apoptotic pathway [19]. Efficacy of ibrutinib was also investigated using a canine model of B-cell lymphoma. There was a 70% reduction in tumor burden. After testing for BTK occupancy, it was determined that a single dose of 2.5 to 20 mg/kg was sufficient to fully occupy BTK [18]. Furthermore, ibrutinib can affect the CLL microenvironment by inhibiting CD40, BAFF, Toll-like receptor and cytokine signaling [20]. The above preclinical findings have led to further clinical trials [21].

#### Clinical studies

##### Chronic lymphocytic leukemia (CLL)

A phase 1 dose escalation study of ibrutinib as a single agent enrolled 56 patients with relapsed/refractory B-cell malignancies, including non-Hodgkin’s lymphoma, CLL, and Waldenstrom’s macroglobulinemia [22,23]. In this study, ibrutinib was well tolerated with substantial activity across B-cell histologies. Fifty-six patients were treated over seven cohorts. Ibrutinib was administered in 1.25, 2.5, 5.0, 8.3, 12.5 and 17.5 mg/kg/day dose orally once per day for 28 days followed by a 7-day rest period to determine maximum tolerated dose (MTD). Pharmacokinetic studies showed that ibrutinib had a mean peak plasma concentration observed at 1–2 hours after administration. Most adverse events were grade 1 and 2 (including neutropenia and thrombocytopenia) and self-limited. Dose limiting events were not observed, even with prolonged dosing. Full occupancy of the BTK active site occurred at 2.5 mg/kg/day, and dose escalation continued to 12.5 mg/kg/day without reaching MTD. The dose of 420 mg daily was chosen for further studies. Objective response rate (RR) in 50 evaluable patients was 60%, including complete response (CR) of 16% and median PFS in all patients was 13.6 months.

With the promising results of phase 1 study in CLL, a phase 1b/II study was done to evaluate the efficacy, safety, pharmacokinetics and pharmacodynamics [24]. A total of 85 patients with relapsed/refractory CLL/SLL received ibrutinib, the first-in-class, oral covalent inhibitor of BTK; Among these 85 patients, 51 received 420 mg and 34 received 840 mg. The overall RR was the same for both groups (71%), and an additional 20% and 15% of patients in the respective group had a partial response with lymphocytosis (PR-L). The response was independent of clinical and genomic risk factors present before treatment, including advanced-stage disease, the number of previous therapies and 17p13.1 deletion. After 26 months of follow-up, the estimated PFS and OS rates were 75% and 83% respectively. Lymphocytosis was found to be treatment related and was not a sign of disease progression. It was thought to be due to lymphocyte mobilization by ibrutinib from bone marrow, lymph nodes, and spleen where stromal elements promote leukemic-cell proliferation, drug resistance, and survival. Another interesting finding from this study was that patients with IgVH unmutated (hence higher risk) had earlier resolution of lymphocytosis and higher response rate (P = 0.02). This was possibly due to the fact that these B cells tend to have higher BCR signaling and therefore more dependence on this pathway [25].

An interim update reported phase Ib/II data from 116 patients who were grouped into three cohorts [26]. First cohort included 31 treatment -naïve patients aged ≥ 65 in which 26 patients received 420 mg/day of Ibrutinib whereas 5 received 840 mg daily. Second cohort comprised relapsed/refractory CLL patients (n = 61), out of which 27 patients received 420 mg of Ibrutinib per day and 34 received 840 mg/day. Third group with high-risk relapsed/refractory (defined as relapse within two years following treatment and/or presence of 17p deletion) received ibrutinib 420 mg (n = 24). For the treatment-naïve CLL patients >65 years old, their median age was 71 years old (age 65–84) and the median follow-up durations for the whole group was 16.6 months (range 1.4-23.2). Unmutated IgVH, 11q deletion, and 17p deletion were found in 55%, 3%, and 7% of patients in this cohort. The overall RR by International Workshop on CLL criteria was 71% (10% CR and 61% PR). Among these, PR-L was 10%. This PR-L, defined as 50% reduction in lymphadenopathy with residual lymphocytosis, is not considered as clinical progression but rather as a form of response called compartmental shift where the drug induces mobilization of leukemic B cells from the bone marrow and spleen into the blood. Overtime, many of the patients had more than 50% decrease in absolute lymphocyte count compared with baseline [26-29]. For the relapsed/refractory CLL cohort, the median age was 64 years old (range 40–81) and the number of prior treatments were four for the whole cohort. There were unmutated IgVH (86%), 17p deletion (37%) and 11q deletion (40%) documented. Median follow up was 17.5 months for patients who received 420 mg and 10.3 months for those who received 840 mg. The overall RR was 67% (CR = 3% and PR 64%). Lastly, for high-risk CLL, all 24 patients received 420 mg of ibrutinib. Median age was 68 years old (range 37–82) with a median of four prior treatments. The documented unmutated IgVH, 17p deletion, and 11q deletion were 83%, 30% and 35%, respectively. The median follow-up was 10.3 months (range 1.1-11.5) and the overall response was 50%, all partial remissions, with 29% achieving PR-L (Table 1). Four percent of patients had progressed while on treatment. The adverse effects reported were grade 1 to 2 including joint pain, nausea, rash, infection, upper respiratory infection, diarrhea and fatigue. Rare hematologic side effects were also observed including neutropenia and thrombocytopenia [26-29]. Lymphocytosis was again found to resolve faster and occur more frequently in CLL patients with unmuted IgVH. The results of these studies also show that although the response rates were high, the CR rate with ibrutinib remained low; therefore, the activity of this drug needs to be evaluated in patients who have received less treatment.

**Table 1:** Ibrutinib in clinical trials for chronic lymphoid leukemia

**Studies**	**Phase Ib/II for CLL/SLL**	**Phase Ib/II for CLL patients (N=116) **[26]			**Ibrutinib**	**Ibrutinib**
					**+**	**+**
					**Rituximab**	**Ofatumumab**
	**(High risk refractory/relapsed)**	**Treatment naïve patients >65 years old**	**Relapsed/refractory**	**High risk relapsed/refractory**	**(Relapsed/refractory CLL)**	**(Relapsed/refractory CLL)**
	**[N=85] **[24]				**[N=40] **[40]	**[N=40] **[41]
ORR	71%	71% (CR=10%, PR=61%)	67 % (CR=3%, PR=64%)	50% (PR=50%, CR=0%)	85%	100%

ORR = overall response rate; CR = complete remission; PR = partial remission.

Single agent ibrutinib is being compared with ofatumumab for R/R CLL (RESONATE trial) [30]. RESONATE-2 will investigate single agent ibrutinib versus chlorambucil as frontline therapy for newly diagnosed elderly patients with CLL [31].

##### Mantle cell lymphoma (MCL)

MCL is characterized by the overexpression of cyclin D1 due to the translocation t (11:14) (q13; q32). Although there are promising treatment modalities, a great majority of patients with MCL remain incurable [32]. Cinar et al. demonstrated a moderate to strong BTK expression in all MCL cases (n = 19) compared to benign lymphoid tissues. Treatment of MCL cell lines (Mino or Jeko-1) with ibrutinib resulted in decreased phospho-BTK-Tyr^223^ expression. Also, ibrutinib inhibited the viability of both Mino and JeKo-1 cells in concentration- and time-dependent manners. Ibrutinib also induced concentration-dependent apoptosis in both cell lines and decreased the levels of anti-apoptotic Bcl-2, Bcl-xL, and Mcl-1 protein. These findings suggest that BTK signaling plays an integral role in MCL cell survival and targeting of BTK is an encouraging therapeutic modality for this type of disease [32,33]. In a phase 1 study of ibrutinib, there is an antitumor activity in several types of NHL including mantle cell lymphoma. A Phase 2 study was performed where ibrutinib was investigated at a daily dose of 560 mg in 111 patients with relapsed/refractory mantle-cell lymphoma [34]. Patients were divided into two groups: 1) those who received at least 2 cycles of bortezomib therapy; 2) those that had received less than 2 complete cycles of bortezomib or had no prior bortezomib therapy. The primary end point was the overall response rate (ORR). Secondary end points were duration of response, PFS, OS and safety. The median age was 68 years old, and 86% of patients had intermediate-risk or high-risk mantle-cell lymphoma according to clinical prognostic factors. A response rate of 68% (75 patients) was observed, with a complete remission rate of 21% and a partial response rate of 47%; prior treatment with bortezomib had no effect on the response rate. Median follow-up was 15.3 months and the estimated median response duration was 17.5 months. The median OS was not reached, the estimated rate of OS was 58% at 18 months. The most common treatment-related adverse effects were mild to moderate diarrhea, nausea and fatigue. Grade 3 or higher hematologic events included neutropenia (16%), thrombocytopenia (11%) and anemia (10%). The study shows that ibrutinib is a durable single-agent effective in relapsed/refractory MCL. Ibrutinib was approved by FDA for relapsed/refractory MCL who at least received one prior therapy. However, the complete response rate is lower than that associated with combination regimens, and it is not yet known whether the addition of ibrutinib can improve the molecular response rate.

##### Diffuse large B cell lymphoma and multiple myeloma

There are early studies of ibrutinib in DLBCL and multiple myeloma. These studies have shown that ibrutinib has cytotoxic effects on these cells and warrants further research. Dasmahapatra et al. examined the interaction of ibrutinib and the proteasome inhibitor (bortezomib) in DLBCL and mantle cell lymphoma cells, including those highly resistant to bortezomib. Co-administration of ibrutinib and bortezomib synergistically increased mitochondrial injury and apoptosis in germinal center or activated B-cell like DLBCL and MCL cells. These events were accompanied by marked AKT and NFkB inactivation, down-regulation of Mcl-1, Bcl-xL and XIAP which enhanced DNA damage and endoplasmic reticulum stress. The same interactions were seen in highly bortezomib-resistant DLBCL and MCL cells and in primary DLBCL cells [35]. On the other hand, Rushworth et al. reported that ibrutinib is cytotoxic to malignant plasma cells from patients with multiple myeloma and the treatment with ibrutinib significantly augments the cytotoxic activity of bortezomib and lenalidomide chemotherapies. It was described that the cytotoxicity of ibrutinib in multiple myeloma is mediated through an inhibitory effect on the NFkB pathway. In addition, ibrutinib blocks the phosphorylation of serine-536 of the p65 subunit of NFkB, preventing its nuclear translocation, leading to down regulation of anti-apoptotic proteins Bcl-xL, FLIP and survivin, culminating in caspase-mediated apoptosis within the malignant plasma cells [36,37].

It has been shown that activated B cell (ABC) but not germinal center B cell (GCB) subtypes of DLBCL cell lines are driven by "chronic active" B cell receptor (BCR) signaling [5,38]. BCR subunit CD79B mutations occur in 21% of ABC but only 5% of GCB DLBCL tumors. In a phase 2 study patients with relapsed/refractory DLBCL received ibrutinib 560 mg daily [39]. Seventy subjects were enrolled; median age was 63 yrs (28–92); median prior systemic therapies 3 (1–7); 23% had prior stem cell transplant. Ibrutinib was well tolerated. In the ABC subtype (N = 29), ORR was 40% (10/25), CR 8% (2/25) and PR 32% (8/25). The median PFS was 5.5 months in ABC responders. PR was observed in only one with the GCB subtype (N = 20) and none in unclassifiable cases (N = 16). Therefore preferential response favored ABC subtype of DLBCL (p = 0.0126), but ibrutinib sensitivity did not require a BCR mutation. These results suggest that future clinical trials of ibrutinib in DLBCL should aim for patients with ABC subtype of DLBCL.

### Ibrutinib in combination therapy

The encouraging results of ibrutinib in treatment naïve and relapse/refractory(R/R) CLL led the investigators to explore combination therapy to determine. Ibrutinib has been studied in combination with rituximab in R/R CLL (n = 40). This combination has an ORR of 85% [40] (Table 1). This was a phase 2 single-center study of 40 high-risk patients at the time of analysis. Patients were treated with ibrutinib 420 mg daily and weekly rituximab (375 mg/m2) for weeks 1–4 (cycle 1), then daily ibrutinib plus monthly rituximab until cycle 6. This was then followed by ibrutinib daily. The median age was 65 (range 35–82) with a median of 2 prior therapies. Among these, 19 patients had del17p or TP53 mutation (4 without prior therapy), 13 patients had del11q. With a median follow up of 4 months, 17 out of 20 patients evaluable for efficacy achieved a partial remission (PR) for an ORR of 85%, and three achieved a PR with persistent lymphocytosis. In this combination trial, the re-distribution lymphocytosis was noted to peak earlier and the duration was shorter than with single-agent ibrutinib. Treatment was well tolerated and induced very high early response rates.

Ofatumumab was also studied with ibrutinib in relapsed/refractory (R/R) CLL (n = 40) with an ORR of 100% [41]. In this report, 27 patients with CLL/SLL following 2 prior therapies received 420 mg daily, in 28-day cycles. Ofatumumab (O) is added at a dose of 300 mg on day 1 of cycle 2, followed by 2000 mg on day 8, 15, and 22 of cycle 2, Day 1, 8, 15, and 22 of cycle 3, and on day 1 of cycles 5–8. This combination had rapid onset of response, low relapse rate and favorable safety profile.

Ibrutinib was combined with bendamustine (B) and rituximab (R) in a phase I study in patients with relapsed/refractory NHL (FL, MZL, MCL, transformed NHL, and DLBCL) [42]. Treatment consisted of standard R 375 mg/m2 day 1, B 90 mg/m2 days 1 and 2, with escalating doses of ibrutinib (280 mg or 560 mg) every 28 days for 6 cycles. Ibrutinib was continued after cycle 6 in responding patients. Pegfilgrastim was used for patients with severe neutropenia. Eleven patients were enrolled, with a median age of 72 (range 45–84) and a median of 3 prior therapies (range 0–10). Nine patients completed two or more cycles of therapy (median 3, range 1–6), of whom 6 received 280 mg and 3 treated with 560 mg of ibrutinib. No DLTs were observed. Severe lymphopenia (64%) was the most common adverse events. Three patients required dose reductions from 280 mg ibrutinib to 140 mg. Bendamustine dose was reduced to 60 mg/m2 in 1 patient for grade 3 thrombocytopenia. ORR was 38% in 8 evaluable patients. There are phase III clinical trials currently ongoing that test the efficacy of BR +/- ibrutinib in R/R CLL patients. There is also a phase 3 study of BR +/- ibrutinib as front-line therapy for MCL (the SHINE trial, MCL3002).

Phase 1b trial assessing the feasibility of incorporating ibrutinib with R-CHOP was updated at 2013 ASCO [43]. In this study, ibrutinib was given as 280, 420, or 560 mg daily in combination with standard R-CHOP. 17 patients were enrolled. At 560 mg dosage, 1 patient had grade 2 gastritis. The phase 2 dose was established at 560 mg daily. The most common adverse event was neutropenia (77%). The response rate was 100% at the time of analysis in 10 patients (7 CR and 3 PR).

### Conclusion and future directions

Novel agents are being studied for CLL and lymphoid malignancies [14,32,44-49]. Ibrutinib as a single agent has brought encouraging results for the targeted therapy of B-cell lymphoproliferative malignancies. Ibrutinib targets B-cell receptor signaling in CLL/MCL cells. Lymphocytosis with ibrutinib therapy is a remarkable clinical phenomenon, and can stay for a prolonged period of time. Additional novel BTK inhibitors (GDC-0834, CC-292, ONO-4059, CNX-774, LFM-A13 and HM-71224) are currently under active investigation [1]. Further research is warranted to assess long-term effects of prolonged use, such as hypogammaglobulinemia, unusual side effects and opportunistic infection [50]. It is also interesting to evaluate the use of BTK inhibitors in peri-transplantation as well as possible maintenance therapy on high-risk patients. Low CR rate from ibrutinib single agent therapy remains a concern. Ibrutinib appears to have activity in myeloma [36,37,51]. Ibrutinib in combination regimens is an intense focus of research in an attempt to improve the response quality.

## Competing interests

The authors declare that they have no competing interests.

## Authors’ contributions

DL and AN were responsible for study design, data collection and drafting the manuscript. All authors have participated in manuscript development, revisions and approved the final manuscript.
